# Research on Mediating Mechanisms and the Impact on Food Provision Services in Poor Areas from the Perspective of Stakeholders

**DOI:** 10.3390/ijerph181910510

**Published:** 2021-10-07

**Authors:** Tianwei Geng, Hai Chen, Di Liu, Qinqin Shi, Hang Zhang

**Affiliations:** 1 College of Urban and Environmental Sciences, Northwest University, Xi’an 710127, China; gengtianwei1002@126.com (T.G.); lcx@stumail.nwu.edu.cn (D.L.); shiqinqin_1314@126.com (Q.S.); 2 Shaanxi Key Laboratory of Earth Surface System and Environmental Carrying Capacity, Northwest University, Xi’an 710127, China; 3 Institute of Land and Urban-Rural Development, Zhejiang University of Finance and Economics, Hangzhou 310018, China; zhrwdl2000@126.com

**Keywords:** food provision services, mediating mechanism, stakeholders, common demand

## Abstract

Exploring and analyzing the common demands and behavioral responses of different stakeholders is important for revealing the mediating mechanisms of ecosystem service (ES) and realizing the management and sustainable supply of ES. This study took Mizhi County, a poverty-stricken area on the Loess Plateau in China, as an example. First, the main stakeholders, common demands, and behavioral responses in the food provision services were identified. Second, the relationship among stakeholders was analyzed. Finally, this study summarized three types of mediating mechanisms of food provision services and analyzed the influence of the different types of mediating mechanisms. The main conclusions are as follows: (1) Five main stakeholders in the study area were identified: government, farmers, enterprises, cooperatives, and middlemen. (2) Increasing farmers’ income is the common demand of most stakeholders in the study area, and this common demand has different effects on the behavioral responses of different stakeholders. (3) There are three types of mediating mechanisms in the study area: government + farmers mediating corn and mutton, government + enterprises mediating millet, and government + cooperatives mediating apples. On this basis, the effects of the different types of mediating mechanisms on variations in food yield, and trade-offs and synergies in typical townships, were analyzed.

## 1. Introduction

Humans are constantly changing ecosystems to obtain more ecosystem services [1,2] and production materials [3,4,5]; among these outputs, food provision services not only support human survival and reproduction [6,7], but also provide basic material conditions for social and economic development [8,9,10]. However, the quantity of food provision services acquired by humans is not only affected by natural conditions, because stakeholders also have an obvious influence on the quantity and structure of food provision services [11,12,13]. In particular, as a result of the growth in the economy and population, the unreasonable use of agricultural resources, and other factors, food safety problems have become increasingly prominent [14,15,16], and changes and instability in the food structure caused by the behavior of stakeholders are becoming increasingly serious [17,18,19], especially in poor areas [20]. Therefore, taking poor areas as an example, it is urgent to analyze the mediating mechanisms of stakeholders on food provision services.

Some scholars have analyzed the sustainability of food provision services [21,22], food provision models [23], food supply safety [24,25], and changes in the food production space [26] from the perspective of stakeholders. In addition, previous studies have emphasized that the identification of stakeholders is highly important [27,28]. The results of Wu and Daw, who identified stakeholders in food provision services, are representative [13,19]. The studies of Wu and Daw provide a reference by which we can identify the main stakeholders of food provision services. However, little attention has been paid to food production in China’s poor areas, and the main stakeholders in these regions are not clear.

“Stakeholder Theory” notes that the term “demand trigger behavior” [29] refers to the goals of stakeholders, and behavior refers to the actions taken by stakeholders to achieve these goals [30,31]. For example, Wu’s research indicated that the demand of farmers is to increase their income, so they choose to grow cash crops with a large market and high prices [19]. Fedele notes that the mediating mechanism is a process in which one behavior is closely connected to another to achieve a common goal [12]. Therefore, analyzing the common demands and behavioral responses is a prerequisite for clarifying the mediating mechanisms.

In analyzing the behavioral responses of stakeholders and the impact on ecosystem services, Quevedo and Ehara’s research is representative and referential. They applied the Drivers-Pressures-State-Impact-Response (DPSIR) model to evaluate the changes in the blue carbon ecosystem and forest timber services from the perspective of stakeholders [32,33]. The DPSIR model enables the expression of interactions between humans and the environment in a simple manner, and to clarify the relationship between society and ecosystems. It can also be used as an analytical method to construct the causal relationship between human activities and ecosystem services [32,33]. Thus, it provides a reference for analysis of the influencing factors and mediating mechanisms of food provision services. For example, drivers and pressures are similar to the demands of stakeholders, and the state, impact, and response reflect the behavioral response of stakeholders. The DPSIR model expresses the analytical thinking with regard to the mediating mechanism of food provision services.

A large amount of research has been conducted on the common demands of stakeholders [34,35], involving environmental changes [35], mental health [36], and public policies [37]. Some scholars have further analyzed the impact of common demands on the collective decision making [38,39], collective behavior, and reciprocal behavior of stakeholders [40]. However, few studies have examined the behavioral responses of stakeholders and the relationships of stakeholders in response to common demands. In addition, the research on stakeholders has mostly adopted qualitative methods and lacks quantitative methods. Some scholars have used the association network method to quantitatively explore the complex interconnections among social phenomena, which provides a reference method for quantitatively exploring relationships among stakeholders [41,42].

Therefore, to analyze the mediating mechanisms and the impact on food provision services in a poor area of China, the following issues must be addressed:

(1) What are the common demands and behavioral responses of stakeholders in food provision services in this region?
(2) What are the relationships among the stakeholders in the region?

First, in this study, we designed a conceptual framework of the mediating mechanisms of food provision services. Second, using Mizhi County, a poverty-stricken area on the Loess Plateau of China, as an example, the main stakeholders of food provision services and their demands and behaviors were identified, and, in the current paper, the similarities and differences in the demands and behaviors of different stakeholders are discussed. Third, the association network method was used to analyze the relationships among stakeholders, and three types of mediating mechanisms of food provision services are summarized. Finally, the effects of different mediating mechanisms on food provision services were analyzed. The results not only reveal the mediating mechanisms of food provision services but also provide a reference for the sustainable supply of food provision services in similar areas.

## 2. Study Area and Methods

### 2.1. Study Area

**Figure 1:**
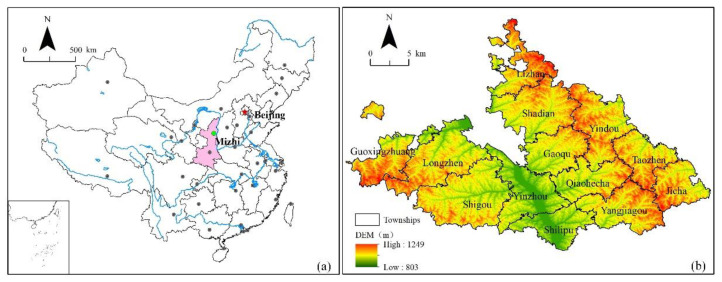
Map of the study area: (**a**) location of Mizhi County, (**b**) Digital Elevation Model (DEM) of Mizhi County.

Mizhi County is located in Yulin City, northern Shaanxi (Figure 1a). It covers an area of 1212 km^2^, contains 13 townships, and has a total population of 224,000 [43]. Mizhi County has a high elevation in the east and west, and a low elevation in the center [44]. The east side is a ridge-shaped hilly area with deep gullies and steep slopes. The west is dominated by the Liangmao landscape, adjacent to a sandy area, and the center is dominated by the Wuding River, with low terrain [45,46]. The overall climate is temperate and semi-arid, with vertical and horizontal ravines, large changes in elevation, low vegetation coverage, and prominent surface fragmentation (Figure 1b).

Mizhi County has closed traffic, scarce resources, and an undeveloped economy. The agricultural population amounts to 184,000, accounting for more than 82% of the total population [47]. At present, the primary industry (agriculture) is still the main source of family income in rural areas. Mizhi County was identified as a national poor county in 2012. The lack of resources and the large number of poor people have become significant challenges for Mizhi County’s development. However, since 2015, Mizhi County has implemented a series of policies to change the food production structure (planting/breeding) to increase farmers’ income with help from the government, cooperatives, enterprises, and other stakeholders. By 2018, Mizhi County had successfully risen above the poverty level. Therefore, this case study provides a good research platform for the analysis of the mediating mechanisms of food provision services under the influence of stakeholders. The term “enterprise” refers to a for-profit organization that engages in economic activities such as food production and other food businesses. A cooperative is the form of economic organization in which farmers voluntarily join forces for production and business cooperation.

Mizhi County has a temperate, semi-arid climate with insufficient rainfall throughout the year and a concentrated rainfall in the summer. In addition, Mizhi County is a typical loess hilly and gully region with an undulating and broken terrain. Due to its climate and topography, Mizhi County has historically been dominated by temperate, drought-tolerant agriculture. Based on the social survey and Statistical Yearbook, this study selected the nine most important food provision services in Mizhi County from 2008 to 2018: corn, millet, potato, green bean, jujube, pear, apple, pork, and mutton.

### 2.2. Methods

#### 2.2.1. The Conceptual Framework of the Stakeholder Mediating Mechanism

**Figure 2:**
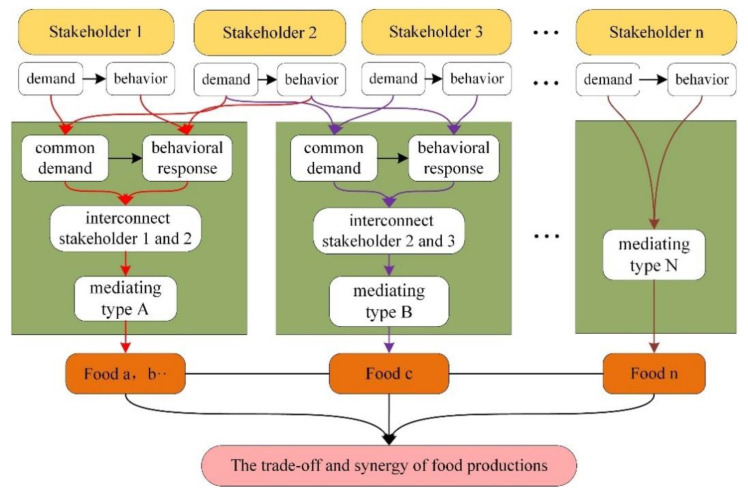
The conceptual framework of the stakeholder mediating mechanism.

This study referred to the Stakeholder Theory and the DPSIR model to design a conceptual framework of the stakeholder mediating mechanism [13,19,29,30,31,32,33], with the aim of showing the formation process and the results of the mediating mechanisms (Figure 2).

The elements of the conceptual framework are stakeholders, common demands and behavioral responses, mediating mechanism types, and results. In the figure, different colored lines represent the formation process of different types of mediating mechanisms. As shown in Figure 2, different stakeholders have different demands, but they also have common demands. Under common demands, different stakeholders have different behavioral responses and establish contact with one or more types of food. For example, stakeholder 3’s demand involves only one type of food, whereas the demands of stakeholder 1 and stakeholder 2 involve many types of food. On this basis, different types of mediating mechanisms are formed, such as the mediation of one food type by many stakeholders (mediation type B) or the mediation of many food types by many stakeholders (mediation type A). Different mediating mechanisms ultimately change the trade-offs and synergies of food provision services. In addition, the framework is only used to reveal the food provision services process, and not the food distribution process.

It should be noted that since 2015, as mentioned above, Mizhi County has implemented a series of policies to change the food production structure, so this study specifically refers to the mediating mechanisms of Mizhi County since 2015.

#### 2.2.2. Identification of Stakeholders

The identification of stakeholders was the basis of the research in this article. The methods of identifying stakeholders mainly include literature analysis [48], face-to-face interviews [49], group discussion [50], and expert identification [51,52]. Compared with other methods, the expert identification method is fast, efficient, and convenient. It is particularly effective in identifying major stakeholders and secondary stakeholders [53,54], and is widely used. Therefore, in this study we used expert identification to identify major stakeholders, as explained in the following paragraph.

First, through social research and a review of the research of existing scholars [13,19,54], we selected nine stakeholders in food provision services. Then, according to the stakeholder assessment index (Table 1), local government officials, technicians from the Bureau of Agriculture, scholars engaged in sociological research, and college teachers familiar with Mizhi County were invited to assign 0–5 points to stakeholders. In the scoring results, the stakeholders with at least three index scores of more than 3 points were identified as the main stakeholders [54].

#### 2.2.3. Determination of Demand and Behavior

This study used participatory assessment to determine the demands and behaviors of stakeholders. Participatory assessment can help investigators objectively understand the target population and accurately and obtain target information [59,60]. This method is often used in social surveys [54,61]. The specific methods include not only questionnaire surveys, direct observations, and semi-structured interviews, but also participatory discussions and detailed interviews [62,63]. We used questionnaires and direct observation to collect information about the demands and behaviors of different stakeholders in food provision services, and we comprehensively considered the interviewees’ educational background, occupation, language expression ability, logical thinking, understanding of food provision services, and other factors. Some interviewees were selected, and a PhD researcher in the research group used a detailed interview method to obtain key information about the process of food provision services. Finally, on the basis of the information collection, the common demands and behavioral responses of the stakeholders were analyzed.

#### 2.2.4. Analysis of the Relationship among Stakeholders

The association network method was used to analyze the relationship among stakeholders. The calculation method is as follows: the Z value represents the network association degree; the larger |Z|, the closer the semantic relationship [64]. To make the analysis results clearer, we filtered the text content, and analyzed only terms related to food provision services, such as farmers, millet, seeds, and technology. The analysis results take a significance level of 0.1 as the criterion. At this significance level, Z = 1.25; that is, when |Z| > 1.25, the correlation between the two semantic words is significant. Moreover, a Z value higher than 3 can be considered to be a strong association, 2–3 is a moderate association, and less than 2 is a weak association. The Z value is calculated as follows:

(1) $$Z=\frac{f_{ij}-E}{\sqrt{S}}$$

where $f_{ij}$ is the actual number of common occurrences of vocabulary *i* and *j* in the same question; E is the expected number of occurrences of vocabulary *i* and *j* in the same problem; and S is the variance in the common occurrence of vocabulary *i* and *j*.

(2) $$E=p_{i}\times p_{j}\times N$$

(3) $$S=p_{i}\times p_{j}\times N\times (1-p_{i}\times p_{j})$$

where $p_{i}$ and $p_{j}$ are the proportions of the number of repetitions of words *i* and *j* in the total sample; *N* = 176, which is the total amount of recorded text.

### 2.3. Data Sources

The data used in this study were taken from the research of the National Natural Science Foundation of China on ecosystem service change and its mediating mechanism; the survey period was 23–25 June 2019 and 24 July–24 August 2019; and the research location was Mizhi County. From 23 to 25 June 2019, the research group visited Mizhi County to conduct a pre-survey, initially via the government departments of Mizhi County, such as the Bureau of Agriculture, the Bureau of Statistics, and the Bureau of Natural Resources. The data relating to land use of Mizhi County, the Mizhi County Statistical Yearbook, and the annual work report of the Bureau of Agriculture were collected. Then, 5 villages were randomly selected, from which 10 households in each village were selected for the pre-survey. Based on the pre-survey results, the questionnaire was revised and improved. Finally, the formal survey was conducted from 24 July to 24 August 2019. The formal survey was divided into three parts. The first part was the questionnaire for individual farmers. The survey area covered all 13 townships in Mizhi County, and 60 villages were randomly selected. The content of the questionnaire is shown in the Supporting Materials, and mainly includes the basic information of farmers and families, planting and breeding information, and food production. A total of 600 questionnaires were recovered, and questionnaires with missing or abnormal data were excluded. A total of 552 valid questionnaires were obtained, with an effective rate of 92%. Second, a PhD researcher in the research group selected the research subjects and conducted in-depth interviews (Section 2.2.3). Please refer to the Supporting Materials for an outline of the interview. The interviewees included farmers, government officials, and heads of enterprises and cooperatives. The content of the interviews mainly related to the change process of food production and stakeholder information. Third, a questionnaire specifically designed for enterprises and cooperatives obtained valid questionnaires from 23 enterprises and 16 cooperatives. The content of the questionnaire included the scale, nature, business content, products, and raw materials of the enterprises and cooperatives. See the Supporting Materials for details. In summary, all of the above research contents focus on the change process of food provision services in Mizhi County and stakeholders’ information. The ultimate purpose in obtaining the above-described data was to analyze the causes of the changes in food provision services in Mizhi County by gaining insight into the demands and behaviors of stakeholders.

## 3. Results

### 3.1. Analysis of Stakeholder Demands and Behaviors

#### 3.1.1. Stakeholder Identification

**Figure 3:**
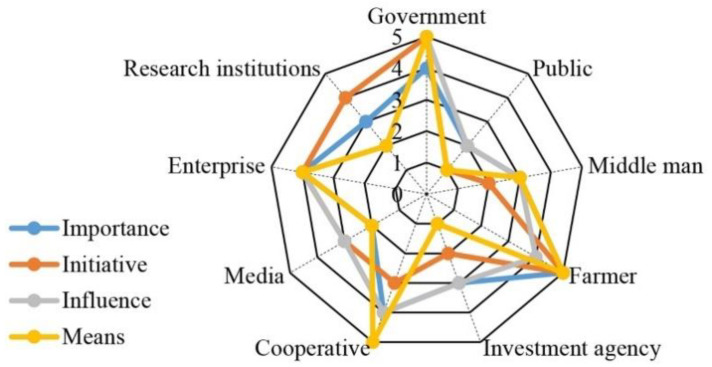
Stakeholder assessment results. Note: Numbers denote stakeholder’ scores.

Using the stakeholder assessment index, we identified five main stakeholders: government, farmers, enterprises, cooperatives, and middlemen, and excluded other stakeholders, such as the public, media, research structures, and investment institutions (Figure 3). Among the included stakeholders, the government and farmers had the highest scores: the importance of the government was 4 points, and that of the other three indexes was 5 points. The influence of farmers was 4 points, and that of the other indexes was 5 points. The overall scores of middlemen were not high, but they scored 3 points in importance, influence, and means; thus, middlemen were also classified as the main stakeholders. The score of each item of enterprises was 4, so they were also considered to be main stakeholders. The initiative of the cooperative was 3 points, the means was 5 points, and the influence and importance were 4 points.

#### 3.1.2. Common Demands and Behavioral Responses of Stakeholders

A total of 552 farmers, 23 enterprises, 16 cooperatives, five middlemen, and six government officials were interviewed, and their demands and behaviors were determined.

It should be noted that, in the analysis, enterprises refer to millet enterprises, because among all of the food processing departments in Mizhi County, millet enterprises are the most numerous and largest, and the millet enterprises play an important role in mediating millet production. Additionally, the number of other types of food enterprise is smaller, and their mediation effect on food production is also smaller. Furthermore, cooperatives refer to apple cooperatives, which, according to our research, are the most influential among all cooperatives in Mizhi County due to the large number of participants, large scale of planting, and high level of government support. Other types of cooperatives, such as potato cooperatives, are mostly stagnant due to low participation of farmers, small scale, backward management, and other factors.

It can be seen that, although the demands of stakeholders are very different, they are similar in some respects (Table 2). The government, farmers, enterprises, and cooperatives all have in common the demand to increase farmers’ income. The reason for this is that companies need to fulfill their social responsibilities, and cooperatives need to increase the income of their members. In addition, poverty alleviation requires the participation of different stakeholders in the entire society. Cooperatives and enterprises have the closest relationship with farmers and can directly increase farmers’ income through their behaviors. Therefore, increasing farmers’ income is a common demand of most stakeholders in the study area.

The behavioral responses of stakeholders under the influence of common demands are both different and closely related (Table 2). For example, to increase farmers’ income, the government and enterprises provide convenient conditions to encourage farmers to plant millet. The government provides seeds and technology for farmers, and helps them establish purchasing relationships with enterprises. Enterprises help farmers through planting bases and purchasing relationships. There are obvious differences in the behaviors of the government and enterprises because different stakeholders have different means of exerting influence.

### 3.2. Relationships among Stakeholders

**Figure 4:**
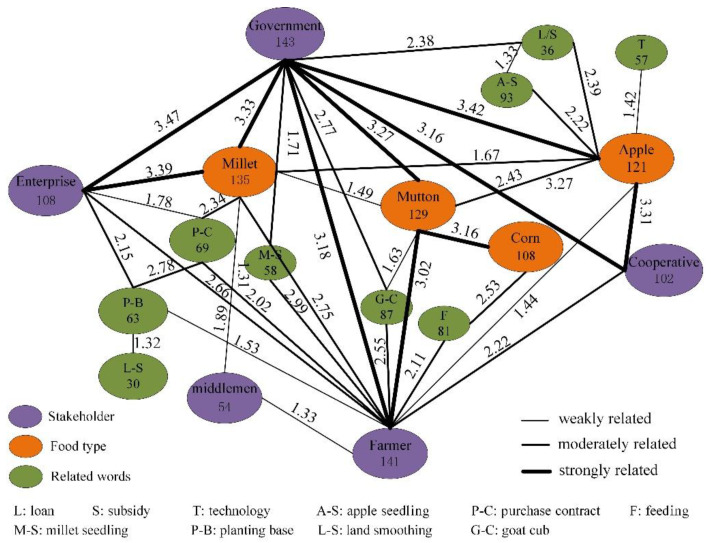
Relationship among stakeholders. Note: There is a detailed explanation of the legend in the Supporting Materials.

The calculation results of the association network are shown in Figure 4. The value on the line is the Z value, and the number in the ellipse represents the total number of times the word appears. The thickness of the line indicates the degree of association, and a thicker line indicates a greater degree of association and a greater role in the association network [64].

As the figure shows, there are complex relationships among stakeholders and food:

(1) The strength of the relationships among stakeholders varies. The results show that government–farmer, government–enterprise, and government–cooperative are strongly related; enterprise–farmer and cooperative–farmer are moderately related; middleman–farmer is weakly related; and the middleman has no relationship with the enterprise, government, or cooperative. All stakeholders, with the exception of middlemen, play an important role in the network of food provision services; however, the government has three strong relationships, which indicates that it plays the most important role in food provision services.

Middlemen play a small role in food provision services, possibly because they have few demands in common with the other stakeholders. In addition, as a result of the improvement of transportation, farmers and enterprises have established more direct and effective contact, and their dependence on middlemen is decreasing.

(2) Stakeholders and food can form strong networks. Figure 4 shows that food and stakeholders can form a closed strong correlation network. This network not only shows the stakeholders who are closely connected to food, but also enables analysis of the role of stakeholders according to the Z value. For example, a strong network with millet production is government–millet–enterprise, which means that, in millet production, the government and enterprise are the main stakeholders, and, according to the Z values, the role of the enterprise is more important than that of the government. In addition, the figure shows two other strong association networks, i.e., government–mutton–farmer and government–apple–cooperative, which indicates that in the production of mutton, the government and the farmer are the main stakeholders, and the role of the government is higher than that of the farmer. In apple production, the government and the cooperative are the main stakeholders, and the role of the government is higher than that of the cooperative.

### 3.3. Mediating Mechanisms and Affects

#### 3.3.1. Types of Mediating Mechanisms

**Figure 5:**
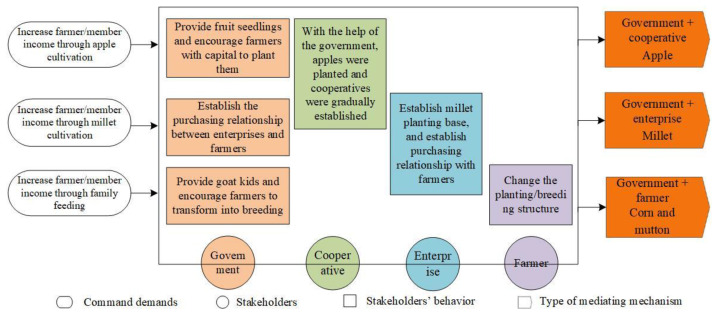
Types of mediating mechanisms. Note: Light orange, olive, green and lavender denote the behavior of government, cooperative, enterprise and farmer respectively. Dark orange denotes the type of mediating mechanisms.

Based on the analysis of stakeholders’ common demand, behavioral response, and the association network of stakeholders, we summarized the three types of mediating mechanism of food provision services in Mizhi County since 2015 and named them according to the stakeholder + food type, i.e., government + farmers mediating corn and mutton, government + enterprises mediating millet, and government + cooperatives mediating apple (Figure 5). From left to right in the figure are the common demands and behavioral responses of stakeholders, and the mediating mechanisms (Figure 5). For example, both the government and cooperative have the demand to increase farmers’ income, which is realized by influencing apple production. Thus, the mediation of the government and cooperative on apple production is formed. In the same manner, the two other types of mediating mechanisms are formed.

The figure also shows that the government plays a key role in the three types of mediating mechanisms. The government not only directly affects farmers’ food production behavior but also indirectly affects farmers’ behavior through cooperation with the enterprise and the cooperative. The cooperative and the enterprise play a key role in the production of millet and apple, respectively, and farmers are the greatest beneficiaries of the mediating mechanisms.

#### 3.3.2. The Effects of Mediating Mechanisms

**Figure 6:**
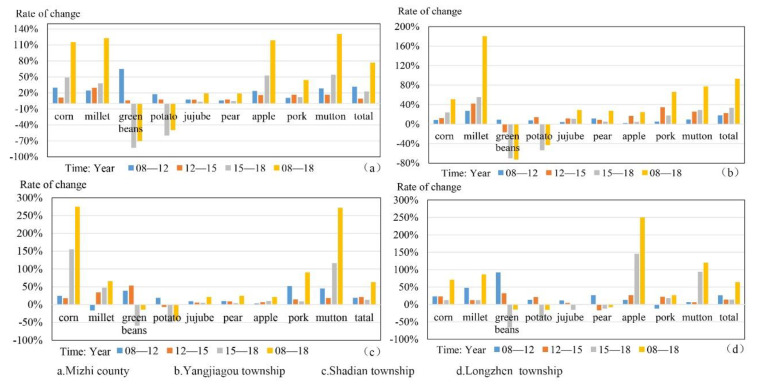
Change rate of food production in Mizhi County and typical towns from 2008 to 2018. Note: The data were taken from the Mizhi County Statistical Yearbook.

To reveal the effects of mediating mechanisms, we analyzed the change characteristics, trade-offs, and synergies of food provision services in Mizhi County (Figure 6; Table 3). Because the yield characteristics of different types of food are different, in this study we adopted the yield change rate to analyze trade-offs and synergies in food provision services. According to the research results, three townships with obvious change characteristics were selected for detailed analysis.

As Figure 6a shows, the food output of Mizhi County in the past 10 years has shown a steady growth trend; however, the characteristics of different types of food differed: the corn, millet, apple, and mutton cumulative growth rates were the highest, at more than 100%; the jujube and pear accumulated growth increment was low; and green bean and potato were the only two types of food with declining yields. In addition, we found that all foods showed an increasing trend before 2015; however, from 2015 to 2018, they showed completely different change characteristics. For example, corn, apple, and mutton showed abnormal and high growth rates, whereas green beans and potato showed a very high decline rate. The change characteristics of various foods in 2015–2018 were not only the main factors causing the overall change characteristics of all kinds of food in the study period, but also directly affected the trade-offs and synergies between foods (Table 3). Among these, the synergistic relationship was concentrated mainly in corn, millet, apple, pork, and mutton, whereas the trade-off relationship was concentrated in green beans and potato.

It is generally believed that as a result of the improvement in farmers’ planting experience and the progress of science and technology, food production will show characteristics of stable or fluctuating growth, so there should be a synergistic relationship between types of food [65,66]. However, the above analysis results show completely different characteristics. Before 2015, the characteristics of various food changes conformed to the abovementioned law. However, from 2015 to 2018, some foods showed abnormally high rates of growth or decline, which directly affected the final food change characteristics. These abnormal changes in food production were the results of mediating mechanisms, and the mediating mechanisms also directly affected the change characteristics and trade-off synergistic relationship of food provision services in Mizhi County from 2008 to 2018.

As shown in Figure 6b and the Supporting Materials, the overall characteristics of food changes in Yangjiagou Township were similar to those in Mizhi County except that the growth rate of millet in Yangjiagou was very high. The trade-offs and synergies were similar to those in Mizhi County. Millet–corn, millet–mutton, and corn–mutton showed high synergies, whereas potato–millet, potato–corn, green bean–millet, and green bean–corn showed high trade-offs. The township represented by Yangjiagou Township belongs to the mediation type of government + enterprises mediating millet.

As shown in Figure 6c and the Supporting Materials, food changes in Shadian Township were similar to those in Mizhi County. The difference is that the growth rates of corn and mutton in Shadian Township were very high. The growth rates of pear, jujube, and apple were all low, so they had a high synergistic relationship with each other. Potato and green bean declined sharply from 2015 to 2018, which also caused the trade-off relationships for potato–millet, potato–corn, green bean–corn, green bean–millet, potato–mutton, and green bean–mutton. The township represented by Shadian belongs to the mediation type of government + farmers mediating corn and mutton.

As shown in Figure 6d and the Supporting Materials, in Longzhen Township, the growth rate of apple was the highest. The overall growth rates of corn, mutton, and millet were high, whereas that of pear, jujube, potato, and green bean was negative. Millet–corn, apple–mutton, apple–corn, and apple–millet showed a synergistic relationship, but the synergistic value was lower than that of other townships. The trade-offs focused on potato, green bean, jujube, and pear. The township represented by Longzhen Township belongs to the mediation type of government + cooperatives mediating apple.

## 4. Discussion

### 4.1. The Importance of Research from the Perspective of Stakeholders

Food production is no longer merely an agricultural activity; it is also increasingly becoming an economic activity [19]. Particularly in poor areas such as Mizhi County, food production has become the most important source of income for families, and the economic characteristics of food production have become obvious. Among the five main stakeholders that we identified in Mizhi County, all stakeholders, with the exception of farmers, have control of market information to varying degrees. Stakeholders will influence and change the type and quantity of food provision service; thus, it is extremely important to analyze food provision services from their perspective.

The most important means to analyze food provision services from the perspective of stakeholders is to analyze the stakeholders’ demands and behaviors, and the associated network among them. Many studies have noted that stakeholders have the same expectations and preferences [67]; however, different stakeholders have different focuses of attention and social resources, and have different impacts on management and decision making [68]. When all stakeholders reach consensus and take action, the production characteristics will change [69], which also provides a more appropriate research perspective for analysis. For example, this study showed that most stakeholders have the demand to improve farmers’ income, and different stakeholders achieved this goal in different ways. The government provides seeds and technology with while companies and cooperatives solve market problems. Therefore, when we clarify the common demand of all stakeholders, it is easier to understand the behavioral responses of stakeholders, and the complex relationships behind food provision services gradually become clear.

The analysis of the mediating mechanisms of food provision services is based on the demands and behaviors of stakeholders; however, the key is to reveal the association network formed by the common demands and behavioral responses of different stakeholders. Different stakeholders have different social resources and rights, so their status in the network differs. In this study, the government had an influence on almost all foods, ranking highest among all stakeholders and playing a leading role. The enterprise, cooperative, and farmer took second place and played a supporting role. The food provision services network is complex; an association network centered on stakeholders who play a leading role can only be constructed by clarifying the status and role of different stakeholders, so that the relationships among stakeholders and between stakeholders and food gradually become clear. This study identified the government as the core of the association network, including government + enterprise, government + cooperative, and government + farmer as three strong association networks. The mediating mechanisms of food provision services in Mizhi County were formed under the above three association networks. Therefore, only by clarifying the status of different stakeholders, and then the association network of food provision services constructed through the leading stakeholders, can we determine the reasons for changes in the structure and quantity of food provision services.

### 4.2. Regional Differences in Mediating Mechanisms and the Causes

There are obvious differences in the types of and reasons for mediating mechanisms in different regions. Yangjiagou Township has long been the largest millet planting area; the farmers have rich experience in planting, and poverty alleviation industries should be tailored to local conditions. While reducing competition, Mizhi County implemented the “one-township, one-industry” policy and continued to promote millet planting in Yangjiagou Township. Finally, Yangjiagou Township has formed the mediation of government + enterprises mediating millet.

Shadian Township is a high-quality area for goat raising. According to the policy of “one township, one industry”, Shadian Township is a typical family feeding area, so the main stakeholders are the government and farmers. However, due to insufficient forage stock in autumn and winter, and because of the expansion in the scale of goat breeding, the shortage of feed has increased. As a result, many farmers have given up their original crops and planted corn instead. Therefore, Shadian Township has formed the mediation of government + farmers mediating mutton and corn.

Longzhen Township has implemented the policy of apple planting because it has the largest share of the poorest population. Apple is a labor-intensive form of agriculture that has significant advantages in promoting farmers’ employment. The government introduced fruit seedlings and helped some farmers grow them. After the expansion, this effort gradually became a cooperative, so the government and cooperatives are the main stakeholders in Longzhen. Longzhen Township has formed the mediation of government + cooperatives mediating apple.

In summary, according to the planting history of Yangjiagou Township, the natural conditions of Shadian Township, and the development status of Longzhen Township, different industrial and development policies have been implemented in different regions, and as a result, these township have formed different types of mediating mechanisms under the influence of different stakeholders.

### 4.3. Validation and Application Prospects of the Framework

The purpose of this study’s conceptual framework was to explain the process and results of stakeholders’ influence on food provision services by analyzing the demands and behaviors of the stakeholders. The research assumption of this framework is that stakeholders have common demands, and under the influence of common demands, behavioral responses are interconnected. The behavior of stakeholders will affect the output and structure of food production. Finally, under the influence of all stakeholders, the trade-offs and synergies of food provision services will be changed. The change process is the mediating mechanism, and the combination of different stakeholders results in different types of mediating mechanisms.

The conclusions of this article also verify the above hypothesis. In Section 3.3.2, it is shown that there are obvious differences in the changes in different food yields between Mizhi County and typical towns, some of which increase substantially, and some of which decrease substantially. The structure of food production has changed, which ultimately changes the trade-off and synergy relationships. When people change planting areas and planting structures, the variation characteristics of different food yields are completely different. Therefore, it can be said that the behavior of stakeholders directly changes the trade-off and synergy relationships of food provision services. Stakeholders change planting structures and planting areas to fulfill their own demands. Some foods are influenced by a single stakeholder, and some foods are influenced by multiple stakeholders simultaneously. When food is related to only one stakeholder’s demand, it is only influenced by that stakeholder, whereas when food is related to multiple stakeholder’s common demands, it is influenced by multiple stakeholders. This illustrates the different types of mediating mechanism. For example, in the conclusion of this article, the government + enterprises mediating millet and the government + cooperatives mediating apple are the mediation type B in the conceptual framework, and the government + farmers mediating mutton and corn is the mediation type A in the conceptual framework. Corn is affected only by farmers, and is the same as mediation type C. Therefore, the research conclusion of this paper describes the generation process and results of the mediating mechanism and verifies the conceptual framework through the analysis of Mizhi County.

Food production is affected by complex socioecological systems, so many research methods are not suitable [70]. The conceptual framework can provide an interactive tool to provide references for broader research [71]. Therefore, establishing a conceptual framework is a useful method for analysis [72]. The framework proposed in this article aims to reveal the mediating mechanism of food provision services. Food provision services are increasingly influenced by stakeholders. In particular, the effectiveness of management policy depends on whether stakeholders can form a common understanding and take concerted action [73,74]. Therefore, the common demands and behavioral responses of different stakeholders have become the most important content of food provision services analysis [75,76]. This framework enables analysis of the stakeholders’ common demands, behavioral responses, the relationship among stakeholders, and their impact on food provision services. This framework also provides a reference for the further study of other relevant fields of stakeholder research.

### 4.4. Recommendations and Limitations of the Study

Increasing poor farmers’ income to address the challenge of poverty is the most important practical significance of this paper. Based on the research conclusions and references, we propose suggestions from the perspectives of food production and stakeholders to ensure the continuous supply of food and the steady increase in farmers’ income.

(1) Food production: Mizhi Millet won the first batch of National Geographical Indications of Agricultural Products in 2008. It was selected as the National Brand Agricultural Product of “One County, One Product” in 2018 [77]. Mizhi Apple was selected among the National Famous and Characteristic Agricultural Products in 2020. Mizhi mountain mutton is well-known in Northwest China. These honors are awarded by the Chinese government, which indicates that the quality and brand of the agricultural products are highly recognized.

Many scholars have proven that honors can strengthen characteristic industries and promote the optimization of the rural industrial structure [78], promote the development of other industries such as tourism [79], expand the visibility of agricultural products, and increase product sales [80]. Therefore, these honors will greatly promote the future development of agricultural products.

Farmers should continue to develop characteristic agriculture. However, because different food types require different labor, capital, and technology, the corresponding groups of farmers should also be different. Millet requires less capital, technology, and labor, so it is suitable for poor farmers or older farmers. Apples have a long capital payback period, and goats have a high cost; therefore, these two types of foods are more suitable for farmers with better conditions. As a result of the continuous development of characteristic agriculture, the income of farmers will significantly increase, creating a virtuous circle that will not only increase the income of farmers, but also ensure the continuous supply of the service.

(2) Stakeholders: This paper cites the Intergovernmental Science-Policy Platform on Biodiversity and Ecosystem Services (IPBES) analysis framework and proposes suggestions from the perspective of stakeholders. The IPBES is an independent scientific body focused on assessing the state of the world’s ecosystem services and biodiversity. IPBES includes four functions: policies; governance; communication and stakeholder engagement; and funding mechanisms. IPBES provides guidance for the transformation of the relationship between humans and the ecosystem [81].

Studies have indicated that a series of policy support efforts is needed to increase food production, such as food crop subsidy programs and incentive schemes for grain-producing regions [82]. Our analysis of Mizhi County further confirmed that the implementation of such policies will change planting structures and increase food production. Therefore, the government should continue to provide preferential policies for agricultural development. After the poverty alleviation policy, the Chinese government continued to implement the Township Revitalization Strategy to ensure that the policy continues to support the development of rural areas. The Township Revitalization Strategy was proposed by the Chinese central government. The Mizhi County government should further refine the content of the policy to guide local farmers.

Some studies on China show that wise local leadership and effective organizational strategies are of vital importance in the governance of rural problems in China [83], and the government should fully utilize its advantages in organizational management. The core stakeholder in the mediating mechanism of food provision services in Mizhi County is the government; other stakeholders have played their respective roles under the liaison of the government. Due to the benefits provided by mediating mechanisms for stakeholders, in the future, a larger number and variety of stakeholders may be interested in participating in food provision services. Therefore, the government should make rational arrangements and unify the organization to ensure that each stakeholder can utilize its own strengths.

The IPBES analysis framework also shows that the communication and participation of stakeholders is required. Stakeholders have cooperative and competitive relationships, so it is necessary for all stakeholders to communicate to avoid conflicts, reduce competition, and expand cooperation.

Economic activities, local entrepreneurship, and social capital are important factors in addressing the decline of rural areas. Moreover, the key to coping with rural problems is capital [84], which is the funding mechanism, and the realization of this function requires the role of the enterprise, which is the main provider of social capital. As a result of the maturity and development of mediating mechanisms, the role of the government will gradually decrease. In the future, the main promoters of the mediating mechanisms of food provision services in Mizhi County may become enterprises and cooperatives. Only when enterprises (cooperatives) increase capital investment can the mediating mechanism play a role in achieving a win–win situation between the stakeholders of enterprises (cooperatives) and farmers.

Farmers are in a weak position in terms of food production. The main means to address this challenge is to choose suitable characteristic agriculture according to their own conditions, and cooperate with other stakeholders and engage in the effective use of government policies and corporate subsidies to increase household income.

In addition to cooperative relationships, competitive relationships also exist among stakeholders [85]. This study focused on analyzing cooperation among stakeholders, while revealing a lack of competition. Only by combining the cooperation and competition of stakeholders will the full role of stakeholders in food provision services become clear.

## 5. Conclusions

This study shows that stakeholders are the key factors that affect food provision services. In Mizhi County, the government, farmers, enterprises, cooperatives, and middlemen are the five most important stakeholders. Different stakeholders have different demands and behaviors; however, under the common demands and behavioral responses of stakeholders, their relationships are affected, and the mediating mechanisms of food provision services are ultimately formed. In the mediating mechanisms, the government is the leader, enterprises and cooperatives play a supporting role, and farmers are the greatest beneficiaries. Finally, in this paper three types of mediating mechanisms are summarized: government + farmers mediating corn and mutton, government + enterprises mediating millet, and government + cooperatives mediating apple. Different types of mediating mechanisms acted on different townships. This caused changes in the food yield and in the trade-off synergistic relationship in different townships to reflect the townships’ unique regional characteristics.

## Figures and Tables

**Table 1:** Stakeholder assessment index.

Index	Index Description
Importance	The importance of different stakeholders in food production differs. Some stakeholders are essential in the process of food production, but the absence of others will not have a great impact on food production.
Initiative	Some stakeholders will take the initiative to exert influence on food production, while others are weak in initiative and even passively affected by food production.
Influence	The same behavior is performed by different stakeholders, but the results differ. Because the influence of different stakeholders is significantly different.
Means	Means can be divided into direct and indirect means. Direct refers to the behavior of stakeholders directly acting on the food production process; other behaviors are considered indirect means.

Note: The scoring standard is, 1 = low influence; 2 = relevant influence; 3 = medium influence; 4 = high influence; and 5 = very high influence. The classification criteria of the index are determined by referring to the research results of relevant scholars [54,55,56,57,58].

**Table 2:** Stakeholders’ demands and behavioral responses.

Stakeholders	Demands	Behavioral Response
Government	Increase farmers’ income, promote poverty alleviation, and ensure that the farmers who experience poverty alleviation do not return to poverty.	The government encourages farmers to change types of planting and breeding through a series of preferential policies: it provides free seeds and technology, promotes millet planting, and helps farmers establish purchasing relationships with enterprises, promotes apple growing and supports farmers with capital for apple growing, and provides goat kids to poor households and key villages to enable family breeding.
Farmers	Increase food production, raise food prices, increase personal and household incomes.	Change the structure of planting and breeding; abandon planting potato, green bean, and other crops, and plant millet. Some farmers grow apples with the support of the government. Many farmers raise goats as a family unit. At the same time, due to the shortage of goat feed, they also start to grow corn that can be used as goat feed.
Enterprises	Increase enterprise income, reduce food purchasing costs, fulfill social responsibilities and increase farmers’ income.	Establish a planting base to increase the purchase of millet, fulfill social responsibilities, establish purchasing relationships with farmers
Middleman	Increase the amount of food purchased; increase personal income.	Encourage farmers to switch to planting millet
Cooperatives	Increase the income of cooperatives, protect the interests of members, and increase members’ income.	Farmers grow apples with government subsidies and gradually transform from large planters to cooperatives, which employ farmers, pay them wages, and provide them with farming experience and technology

**Table 3:** Trade-off synergy relationship of grain yield change in Mizhi County from 2008 to 2018.

	Corn	Millet	Green Bean	Potato	Jujube	Pear	Apple	Pork	Mutton
corn	1								
Millet	0.95 *	1							
green bean	−0.63 *	−0.57 **	1						
Potato	−0.69 *	−0.57 **	0.96 *	1					
Jujube	0.83	0.92	−0.24	−0.23	1				
Pear	0.87	0.97	−0.41	−0.38	0.98 *	1			
Apple	0.99 **	0.96 *	−0.69	−0.73	0.82	0.28 *	1		
Pork	0.78 *	0.78 *	−0.48 *	−0.44	0.96	0.95	0.91	1	
Mutton	0.99 **	0.97 *	−0.65	−0.71	0.84	0.89	0.99 **	0.91 *	1

Note: * and ** denote statistical significance at 5% and 1%, respectively.

## Data Availability

Data is contained within the Supplementary Materials.

## Supplementary Materials

The following are available online at https://www.mdpi.com/article/10.3390/ijerph181910510/s1, Table S1: Basic information of the farmers; Table S2: Land resources and planting/breeding information; Questionnaire S1: Detailed interview content for farmers and middlemen; Questionnaire S2: Detailed interview content for middlemen; Questionnaire S3: Detailed interview content for Government officials; Questionnaire S4: Detailed interview content for corporate and cooperative; Table S3: The trade-off and synergy of changes in food production in Yangjiagou Township; Table S4: The trade-off and synergy of changes in food production in Shadian Township; Table S5: The trade-off and synergy of changes in food production in Longzhen Township; Figure S1: Mediation types of food provision services in Mizhi County.
