# Supplementary material for: Research on Mediating Mechanisms and the Impact on Food Provision Services in Poor Areas from the Perspective of Stakeholders
Source: Int J Environ Res Public Health. 2021 Oct 7;18(19):10510. doi: 10.3390/ijerph181910510 (PMC8508073; doi:10.3390/ijerph181910510)
Supplement: Supplementary file 1 [file ijerph-18-10510-s001.zip › ijerph-1312030-supplementary.pdf]

International Journal of
Environmental Research and Public Health

MDPI

Supporting Materials A: Research content designed to analyze food provision services in Mizhi County

Table S1: Basic information of the farmers

Gender：	Age:	Family population:	Annual income:
Length of residence：	Agricultural population：	Migrant workers population:	
Planting income：	Farming income：	Fixed salary：	
Subsidy income：	Cooperative：Yes/No	Poor households：Yes/No	
Education level:<br>①Illiteracy<br>②Primary school<br>③Junior high school<br>④Senior high<br>⑤University	Family income sources:<br>①Plant ②Breed<br>③Both above<br>④Service industry<br>⑤Government-affiliated institutions<br>⑥Other	Physical health:<br>①Very health<br>②Healthy<br>③Medium<br>④Unhealthy<br>⑤Very unhealthy	

Table S2: Land resources and planting/breeding information

**Type**	cultivated land	irrigable land	sloping land	leased farmland	terrace	dam land
Area (2015)						
Area (2018)						
Reason for change						
**Type**	number of plots	grapes	apple	pear	jujube	peach
Area (2015)						
Area (2018)						
Reason for change						

**Type**	corn	wheat	millet	scallion	sorghum	potato	sweet potato	green beans
Area (2015)								
Area (2018)								
Reason for change								

**Type**	goat	cattle	pig	fowl	cow	donkey
Number (2015)						
Number (2015)						
Reason for change						

Questionnaire S1: Detailed interview content for farmers and middlemen

1.How long have you been in plantation/breeding？ Have you changed the planting/breeding type in recent years?

2.Please introduce the process, reason and time of planting/breeding type transformation in detail.

3.Are there subsidies for farmers' planting/breeding (yes/no) and subsidy standards ______

4.Planting/cultivation change process (time/scale/yield)

5.What factors will affect/change farmers' planting willingness

6.Main implementation methods and content of government policies (interview): beautiful villages/precise poverty alleviation/rural revitalization.

7.Have the key stakeholders of food production changed in recent years? Why the change?

8.Did the various stakeholders help you grow? Who are these stakeholders?

9.Are there any connection between the various stakeholders?

10.Do you feel you are a major stakeholder in food production? What are your demands and behaviors？

11.Which stakeholder do you think helps the most? Or which stakeholders would you most like to get help from?

12.Do you have anything to add about how and why the food production structure changed?

13.Do you have anything to add to the information about stakeholders in food production?

Questionnaire S2: Detailed interview content for middlemen

1.How many years have you been working as a middleman?

2.What are the main types of food you buy and sell?

3.Which foods do you prefer to buy and sell? Has it changed in recent years?

4.Where is your main acquisition scope?

5.Do you think there is any change in the farmer's planting structure?

6.What is the reason for the change?

7.Who do you think are the stakeholders in food production? And why?

8.Do you feel you are a major stakeholder in food production? What are your demands and behaviors？

9.Do you have anything to add about how and why the food production structure changed?

10.Do you have anything to add to the information about stakeholders in food production?

Questionnaire S3: Detailed interview content for Government officials

1.What is the structure of food production in Mizhi County? What is the largest proportion?

2.Has the food production structure changed in Mizhi County in recent years? What has changed?

3.What is the cause of these changes?

4.What type of crops do you think farmers are more willing to grow? What type of crops are companies more willing to purchase?

5.Are there any government policies or subsidies for food production？ such as beautiful villages/precise poverty alleviation/rural revitalization.

6.What are the main stakeholders in food production in Mizhi County?

7.What are the demands and behaviors of each stakeholder? What is the connection between what they want and what they do?

8.Have the key stakeholders changed in recent years? Why the change?

9.What type of food production does the government value most? Is there any policy in place to help farmers increase production?

10.Do you think the government is a major stakeholder? What are the demands and actions of the government?

11.Do you have anything to add about how and why the food production structure changed?

12.Do you have anything to add to the information about stakeholders in food production?

Questionnaire S4: Detailed interview content for cooperative and enterprise

Name of cooperative____________ Location____________ Time____________ Tel___________

cooperative nature____________ Business type____________

1.Planting / Breeding type of cooperatives __________________

①millet ②corn ③apple ④scallion ⑤jujude ⑥Potato ⑦bean ⑧sorghum

⑨chicken ⑩pig ⑪goat ⑫ cow ⑬other

2.Scale of cooperatives ____________ （Area / quantity / number of households）

3.Types of cooperatives ________

①Farmers organize themselves ②Farmer enterprise alliance

③Farmer government coalition ④Other

4.Membership of cooperatives ______________

①neighborhood- friend ②Farmers in the village ③Farmers in other villages ④Other

5.Main business contents of cooperatives ______________

①Planting / Breeding ②Unified purchase of fertilizer / feed

③Centralized collection and sale of farmers' products ④Subsidies and compensation

6.(Yes /No) will purchase the grain / raw materials of farmers, and the way of purchase ________

①Purchase at the farmer's house ②Farmers sell at Cooperatives

③Fixed sales target ④Other

7.Purchasing farmers from ________

①village②Surrounding villages③Surrounding town④Surrounding county⑤Other

8.(Yes /No) it will organize the sales of grain / raw materials for farmers, and the mode of sale __

①Market sale ②Through middlemen ③Go out and sell

④The merchant orders to the village ⑤Fixed sales target ⑥Other

9.What is the relationship between cooperatives and farmers?

10.Do you feel you are a major stakeholder in food production?

11.What are your demands for food production? What actions will you take to achieve your demands?

12.What are the main stakeholders in food production in Mizhi County? Are there any connection between the various stakeholders?

13.Do you have anything to add to the information about stakeholders in food production?

Name of enterprise ____________ Location____________ Time____________ Tel____________

Enterprise nature____________ Business type____________

1.Main business ________________

①The raw material processing ②Raw material storage

③Raw materials for sale ④Raw material transfer

2. Scale of operation ________(Number of employees)________(turnover)

3. Nature of enterprise ________

①private ②Public ③Public-private partnerships ④State-owned ⑤others

4. Purchased raw material type __________________

①millet ②corn ③apple ④scallion ⑤jujude ⑥Potato ⑦bean ⑧sorghum

⑨chicken ⑩pig ⑪goat ⑫ cow ⑬other

5.The way of purchase ________

①Purchase at the farmer's house ②Farmers sell at enterprise ②Through middlemen

③Fixed sales target ④Other

6. Acquisition of raw material objects ________

①personal ②cooperative ③retailers④Other enterprises ⑤Other

7.Raw material comes from ________

①village②Surrounding villages③Surrounding town④Surrounding county⑤Other

8.Way to sell products________

①Fixed sales target ②retailers ③Go out and sell

④Through middlemen ⑤Customers come to the enterprise to order ⑥Other

9.Address of Sales Target ________

①Surrounding villages②Surrounding town③Surrounding county④Other

10.Compared with before, has there been any change in the way the company operates?

11.What's the change? What is the reason for the change?

12.What type of crops do you think farmers are more willing to grow?

11Do you feel you are a major stakeholder in food production?

13.What are your demands for food production? What actions will you take to achieve your demands?

14.What are the main stakeholders in food production in Mizhi County? Are there any connection between the various stakeholders?

15.Do you have anything to add to the information about stakeholders in food production?

Supporting Materials B:

In Figure 4, we use ellipses with different colors to represent nouns with different attributes. Purple represents stakeholders, orange represents food, green represents nouns related to food production, such as technology, subsidies, apple seedling, etc. Use the thickness of the line to distinguish the strength of the connection between nouns, the thicker the line, the higher the frequency of the common occurrence of the two nouns, and the closer the relationship between them.

Supporting Materials C: The trade-off and synergy of changes in food production in Mizhi County and three townships

Table S3. The trade-off and synergy of changes in food production in Yangjiagou Township

	corn	millet	green bean	potato	jujube	pear	apple	pork	mutton
corn	1								
millet	0.99**	1							
green bean	-0.96*	-0.95**	1						
Potato	-0.89**	-0.85**	0.71*	1					
Jujube	0.31	0.38	-0.13	0.41	1				
Pear	0.22	0.29	-0.23	0.49	0.59*	1			
Apple	0.32*	0.31	-0.54	-0.53	0.40*	0.49	1		
Pork	0.52	0.47*	-0.31	0.11	0.61	0.59	0.69*	1	
Mutton	0.93*	0.92**	-0.91	-0.49	0.55	0.46	0.29	0.66**	1

Note: * and ** indicate significant at the significance level of 5% and 1% respectively.

Table S4. The trade-off and synergy of changes in food production in Shadian Township

	corn	millet	green bean	potato	jujube	pear	apple	pork	mutton
corn	1								
millet	0.89*	1							
green bean	-0.23*	-0.3*	1						
Potato	-0.31*	-0.45*	0.84**	1					
Jujube	0.74	0.54	-0.56	-0.61	1				
Pear	0.43	0.72	-0.05	-0.63	0.87*	1			
Apple	0.57	0.31	-0.44	-0.36	0.87*	0.83*	1		
Pork	0.88*	0.24	0.82	0.32	0.13	0.14	0.11	1	
Mutton	0.99**	0.83*	-0.42*	-0.51	0.79	0.43	0.61	0.56*	1

Note: * and ** indicate significant at the significance level of 5% and 1% respectively.

Table S5. The trade-off and synergy of changes in food production in Longzhen Township

	corn	millet	green bean	potato	jujube	pear	apple	pork	mutton
corn	1								
millet	0.88*	1							
green bean	-0.31	-0.31	1						
Potato	-0.69	-0.41	0.73*	1					
Jujube	-0.58	0.18	0.87**	0.94	1				
Pear	-0.63	-0.15	0.86	0.46	0.63*	1			
Apple	0.78*	0.73*	0.14	-0.35	-0.26*	-0.31*	1		
Pork	0.89	0.62	-0.51	-0.45	-0.47	-0.86	0.73*	1	
Mutton	0.99	0.72	-0.26	-0.65	-0.53	-0.61	0.91**	0.78*	1

Note: * and ** indicate significant at the significance level of 5% and 1% respectively.

Supporting Materials D:

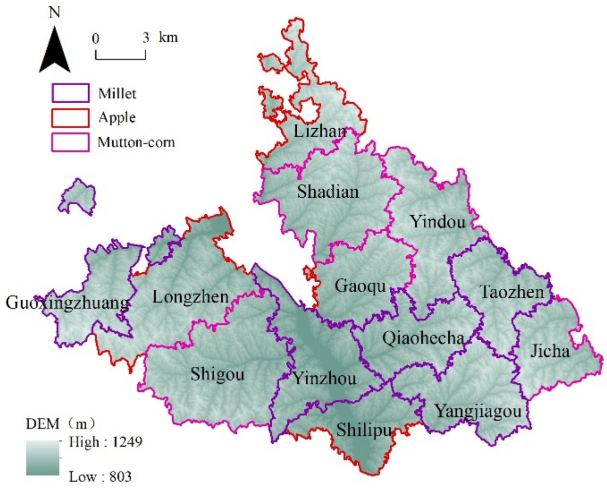

Figure S1. Mediation types of food provision services in Mizhi County
